# PROMOTING HONG KONG ADULTS TO AGE WELL: INTERVENTION EFFECTS ON AGING PREPARATION ACROSS FOUR DOMAINS

**DOI:** 10.1093/geroni/igad104.2181

**Published:** 2023-12-21

**Authors:** Hongmei Lin, Verona Ji Ying Leung, Courtney Sen Yee Yue, Helene Fung

**Affiliations:** The Chinese University of Hong Kong, Hong Kong, Hong Kong; The Chinese University of Hong Kong, Hong Kong, Hong Kong; The Chinese University of Hong Kong, Hong Kong, Hong Kong; The Chinese University of Hong Kong, Hong Kong, Hong Kong

## Abstract

Preparations for old age benefit physical and psychological health in later life. The population in Hong Kong has the longest life expectancy in the world. However, Hong Kong residents prepare less for their old age than their counterparts in the US and Europe. Hence, an evidence-based intervention program was developed to improve old-age preparation in Hong Kong. The program aimed to comprehensively cover the preparation for old age by involving four domains (finance, health, social relationship, and leisure activity). Specifically, the program started with four workshops where participants learned from experts on how to prepare for old age in four domains. Then, participants were grouped according to their domains of interest, and guided planning was provided to empower them to hold their old age-preparation activities for six months. A tracking survey was conducted every two weeks during the period. Finally, 140 adults (31 males; 45-83 years of age, Mage = 65.21, SD = 7.52) finished the intervention program. Findings showed that participants increased their levels of self-control, readiness for, and satisfaction with aging preparation in three domains (all except finance) after joining the workshop. After six months of intervention, a reduction in anxiety and an improvement in meaningfulness were also found in all four domains. These findings suggest that the intervention program motivates individuals to engage more in age-related preparatory behaviors across domains. This has practical implications for training old-age preparation.

